# The Need for Appendicectomy after Appendicular Abscess

**Published:** 1909-08-28

**Authors:** 


### THE NEED FOR APPENDICECTOMY AFTER APPENDICULAR ABSCESS.

The older teaching was that in cases of perityphlitic or appendicular abscess there is no need to do more than open and drain the abscess, the general belief being that the vermiform appendix in these cases either sloughs off and disappears spontaneously, or else becomes fibrosed and obsolete, so as to be no longer a likely seat of disease. There has of late been a growing school that advocates emphatically the need for removing the appendix surgically in all these cases, instead of leaving its future behaviour to chance, for in a large percentage of cases of appendicular abscess that have been incised, drained, and apparently cured there has been subsequent appendicular trouble of one kind or another—avoidable if appendicectomy had been performed at or soon after the original operation upon the abscess. Mr. Battle, having analysed a large number of cases expresses this view as follows:—

The statements of those who held that the appendix is obliterated by suppuration around it are erroneous; it is never obliterated but remains the subject of gross disease which in many instances (20 out of 70) gives rise to a return of symptoms within a short period after the closure of the abscess. The lesions found in those cases in which the appendix has been removed for a return of symptoms do not differ in their character from those found when it has been removed as a matter of routine without waiting for symptoms. Compared with a series of appendices removed for recurring attacks, without evidence of suppuration, the lesions found after removal following abscess are more gross and give fewer instances of apparent recovery from disease. Removal of the appendix after closure of the abscess is a safe procedure, and is not followed by any weakness of the abdominal wall if done in the way suggested (*i.e.* by displacement of the rectus after incision of the anterior part of its sheath, followed by section of the posterior part of the sheath). Moreover, operation at an early date after closure of the abscess enables the surgeon to treat dangerous adhesions and deal with any pelvic disease which may complicate the condition.

